# Shed urinary ALCAM is an independent prognostic biomarker of three-year overall survival after cystectomy in patients with bladder cancer

**DOI:** 10.18632/oncotarget.13546

**Published:** 2016-11-24

**Authors:** Shanna A. Arnold Egloff, Liping Du, Holli A. Loomans, Alina Starchenko, Pei-Fang Su, Tatiana Ketova, Paul B. Knoll, Jifeng Wang, Ahmed Q. Haddad, Oluwole Fadare, Justin M. Cates, Yair Lotan, Yu Shyr, Peter E. Clark, Andries Zijlstra

**Affiliations:** ^1^ Department of Veterans Affairs, Nashville, TN, USA; ^2^ Department of Pathology, Microbiology and Immunology, Vanderbilt University Medical Center, Nashville, TN, USA; ^3^ Center for Quantitative Sciences, Vanderbilt University Medical Center, Nashville, TN, USA; ^4^ Department of Cancer Biology, Vanderbilt University Medical Center, Nashville, TN, USA; ^5^ Department of Cell and Developmental Biology, Vanderbilt University Medical Center, Nashville, TN, USA; ^6^ Department of Statistics, National Cheng Kung University, Taiwan; ^7^ Meharry Medical College, Nashville, TN, USA; ^8^ Department of Urology, The Fifth People's Hospital of Shanghai, Shanghai, China; ^9^ Department of Urology, The University of Louisville, Louisville, KY, USA; ^10^ Department of Urology, The University of Texas Southwestern Medical Center, Dallas, TX, USA; ^11^ University of California San Diego, La Jolla, CA, USA; ^12^ Vanderbilt Ingram-Cancer Center, Vanderbilt University Medical Center, Nashville, TN, USA; ^13^ Department of Urology, Vanderbilt University Medical Center, Nashville, TN, USA

**Keywords:** ALCAM, bladder cancer, transitional cell carcinoma, urothelial cell carcinoma, metastasis

## Abstract

Proteins involved in tumor cell migration can potentially serve as markers of invasive disease. Activated Leukocyte Cell Adhesion Molecule (ALCAM) promotes adhesion, while shedding of its extracellular domain is associated with migration. We hypothesized that shed ALCAM in biofluids could be predictive of progressive disease. ALCAM expression in tumor (*n* = 198) and shedding in biofluids (*n* = 120) were measured in two separate VUMC bladder cancer cystectomy cohorts by immunofluorescence and enzyme-linked immunosorbent assay, respectively. The primary outcome measure was accuracy of predicting 3-year overall survival (OS) with shed ALCAM compared to standard clinical indicators alone, assessed by multivariable Cox regression and concordance-indices. Validation was performed by internal bootstrap, a cohort from a second institution (*n* = 64), and treatment of missing data with multiple-imputation. While ALCAM mRNA expression was unchanged, histological detection of ALCAM decreased with increasing stage (*P* = 0.004). Importantly, urine ALCAM was elevated 17.0-fold (P < 0.0001) above non-cancer controls, correlated positively with tumor stage (*P* = 0.018), was an independent predictor of OS after adjusting for age, tumor stage, lymph-node status, and hematuria (HR, 1.46; 95% CI, 1.03–2.06; *P* = 0.002), and improved prediction of OS by 3.3% (concordance-index, 78.5% vs. 75.2%). Urine ALCAM remained an independent predictor of OS after accounting for treatment with Bacillus Calmette-Guerin, carcinoma *in situ*, lymph-node dissection, lymphovascular invasion, urine creatinine, and adjuvant chemotherapy (HR, 1.10; 95% CI, 1.02–1.19; *P* = 0.011). In conclusion, shed ALCAM may be a novel prognostic biomarker in bladder cancer, although prospective validation studies are warranted. These findings demonstrate that markers reporting on cell motility can act as prognostic indicators.

## INTRODUCTION

Bladder cancer (BCa) is the 9th most common cancer world-wide [1] and 4th most common in men in the USA with an estimated 74,000 new cases in 2015 [2]. Approximately 20–30% of BCa is diagnosed as muscle invasive (MIBC) while 10–30% of patients with non-muscle invasive BCa (NMIBC) progress to invasive disease [3, 4]. While surgical resection of the bladder (cystectomy) can be curative, approximately 50% of cystectomy patients recur with metastases within two years [5]. The risk of progression and recurrence necessitates frequent follow-up, invasive monitoring, and repeated clinical interventions, which decreases quality of life and makes lifelong management of BCa more costly than any other cancer [6]. Moreover, despite proven survival benefit, neoadjuvant chemotherapy is under-utilized in this aging patient population with multiple comorbidities [7, 8]. Prognostic indicators could identify patients likely to benefit from aggressive intervention and improve patient care but there are currently no accurate, non-invasive ways to predict recurrence and monitor treatment response.

Accessibility makes fluid-based biomarkers attractive candidates for the diagnosis and prognosis of BCa [9]. Unfortunately, only a small proportion of fluid-based biomarkers have been investigated for prognostic significance in BCa, with the majority of studies focused on diagnostics [10, 11]. A recent multiplatform genomic analysis highlights the molecular heterogeneity of bladder cancer [12] and underscores the diversity of oncogenic mechanisms that can drive bladder cancer. However, post-translational modifications that universally support malignant progression, such as proteolytic products of cell motility, are promising biomarkers that may act as global predictors of patient outcome regardless of the underlying genetics [13]. Activated Leukocyte Cell Adhesion Molecule (ALCAM) is a cell surface protein capable of homotypic cell-cell adhesion [14–17], the disruption of which, contributes to both normal cell migration and the metastatic dissemination of tumor cells [18, 19]. ALCAM-mediated adhesion is disrupted when its ectodomain is shed by ADAM17 from the surface of tumor cells during malignant transformation [20–22]. Consequently, ALCAM shedding is a molecular indicator of a cellular activity that will ultimately present itself pathologically as invasive and/or disseminated disease. Indeed, we have recently demonstrated that, through preclinical studies of prostate cancer and clinical correlation in colorectal cancer, ALCAM contributes directly to cancer metastasis [22] and histological detection of intra-tumoral ALCAM shedding is prognostic of disease-specific survival in stage II disease [23].

ALCAM has significant potential as a fluid-based biomarker because the shed ectodomain of ALCAM is released into adjacent biofluids. While elevated serum levels of ALCAM have been reported for several non-urothelial neoplasms [20, 24–28], high baseline levels of circulating ALCAM prevent its global implementation as a blood-based biomarker. However, studies of ALCAM in ascites fluid from patients with ovarian carcinomatosis suggest ALCAM in tumor-adjacent fluids, other than blood, could predict outcome [23, 24]. In the bladder, ALCAM expression is restricted to the umbrella cells and several layers of the urothelium, which are in direct contact with the urine. Therefore, we hypothesized that elevated levels of urinary ALCAM would be indicative of invasive tumor progression and, thus, serve as a prognostic biomarker in BCa. Using retrospective cohort studies, we compared the ability of ALCAM gene expression (mRNA), tissue expression (protein), and shedding (blood and urine) to predict overall survival in BCa. This is the first study to provide a multi-level assessment of ALCAM prognostication in cancer and definitively show that it is post-translational processing of ALCAM, defined as ALCAM “shedding”, that is most predictive of patient outcome.

## RESULTS

### ALCAM gene expression

**Figure 1:**
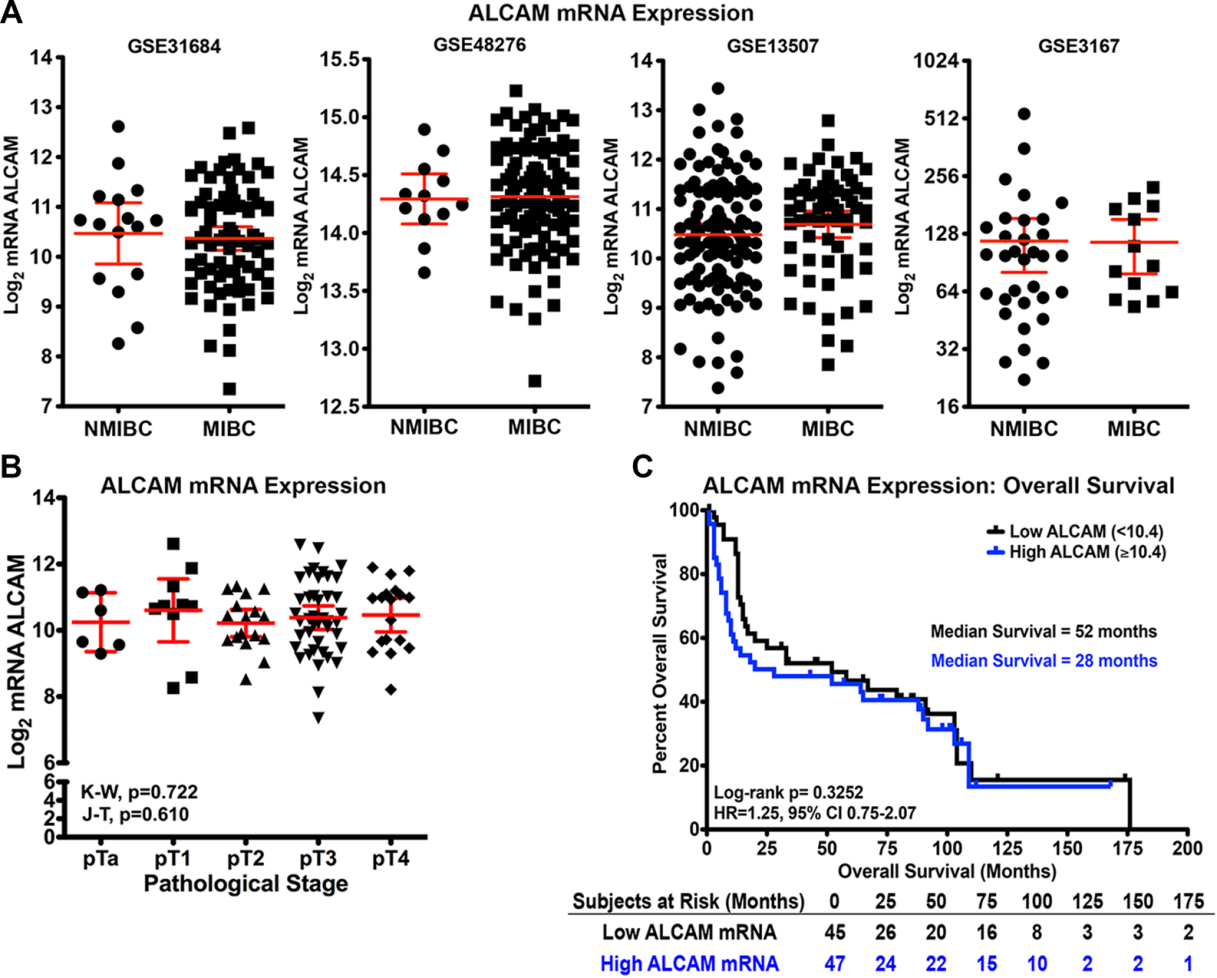
Correlation of ALCAM mRNA with tumor progression and overall survival in bladder cancer (**A**) Gene expression analyses performed on four independent bladder cancer cohorts, GSE31684 (*n* = 93), GSE48276 (*n* = 126), GSE13507 (*n* = 176), and GSE3167 (*n* = 46), available as GEO datasets on NCBI Gene Expression Omnibus comparing Log_2_ mRNA ALCAM levels of non-muscle invasive (NMIBC) and muscle invasive (MIBC) bladder cancer [29–32]. Mean and 95% confidence intervals displayed. (**B**–**C**) Analysis of the GSE31684 dataset for ALCAM Log_2_ mRNA correlation with tumor stage (B) and overall survival (C). (B) Mean and 95% confidence intervals displayed. K-W, Kruskal-Wallis test. J-T, Jonckheere-Terpstra test for trend. (C) Kaplan-Meier curves and Log-rank test for significance of ALCAM dichotomized high/low around the median Log_2_ mRNA level (10.4). HR, Hazard Ratio. CI, Confidence Interval.

Analysis of ALCAM mRNA expression was performed on four independent bladder cancer cohorts available as GEO datasets at NCBI (GSE31684, *n* = 93; GSE48276, *n* = 126; GSE13507, *n* = 176; GSE3167, *n* = 46) [29–32]. A comparison of non-muscle invasive (NMIBC) and muscle invasive (MIBC) bladder cancer revealed that ALCAM expression is not significantly altered during BCa progression to invasive disease (Figure 1A).

To further determine if ALCAM mRNA expression correlated with outcome in BCa, we performed a detailed statistical analysis of the GSE31684 dataset [32]. ALCAM mRNA expression did not correlate with tumor stage (Kruskal-Wallis (K-W), *P* = 0.722; Jonckheere-Terpstra test for trend (J-T), *P* = 0.610; Figure 1B), nor did it significantly stratify patient outcome of overall survival when dichotomized around the median log2 mRNA level of 10.4 (Log-rank, *P* = 0.325; Hazards Ratio (HR), 1.25; 95% Confidence Interval (CI), 0.75–2.07; Figure 1C). Furthermore, multivariable Cox regression analysis reveals that ALCAM gene expression fails to reach significance as an independent predictor of 3-year overall survival after adjusting for available covariates including age, gender and tumor stage (Table 1 Top; adjusted HR, 1.26; 95% CI, 0.94–1.68; *p* = 0.118). Since ALCAM mRNA levels remain unaltered during tumor progression in four independent patient cohorts and fail to predict overall survival by univariable and multivariable analyses, we conclude that ALCAM gene expression is not a viable biomarker for BCa prognosis.

**Table 1:** Assessment of ALCAM mRNA and protein expression as a predictor in a multivariable Cox regression analysis of 3-year overall survival in bladder cancer

Variable	Hazard Ratio	95.0% CI	Significance	Bootstrap Significance
Age (Years)	1.01 ^a^	0.98–1.04	0.404	0.384
Gender	1.32 ^a^	0.70–2.48	0.396	0.383
Tumor Stage	2.46 ^a^	1.69–3.58	< 0.0001	0.001
ALCAM Log2 mRNA	1.26 ^a^	0.94–1.68	0.118	0.107
Age (Years)	1.03 ^a^	1.01–1.05	0.01	0.024
Gender	0.78	0.50–1.21	0.265	0.304
Tumor Stage	1.30 ^a^	1.12–1.50	0.001	0.001
N Stage	1.42	1.10–1.82	0.006	0.017
ALCAM (% Thresholded Area)	1.00 ^a^	0.99–1.02	0.843	0.792

Assessment of ALCAM mRNA (top) and protein (bottom) expression as a predictor of 3-year overall survival by multivariable Cox regression analysis. The mRNA expression cohort is a publicly available dataset at NCBI Gene Expression Omnibus (GEO) [GSE31684] [32]. The ALCAM protein expression was measured by fluorescence intensity (% thresholded area) in the VUMC bladder cancer TMA cohort as described in Table 2. Hazard Ratio is the adjusted hazards ratio for ^a^ every 1 unit increase such as 1 year or 1%. CI, confidence interval. Bootstrap significance is two-tailed with 1000 iterations and a Mersenne twister of 2,000,000.

### ALCAM protein expression

Post-translational proteolytic processing of ALCAM can create a disparity between gene expression and the availability of ALCAM protein. Indeed, ALCAM protein levels frequently fail to correlate with gene transcription [33]. In addition, histological detection of ALCAM has been shown to correlate with disease progression and patient outcome in several non-urothelial cancers [26, 27, 33–38].

**Figure 2:**
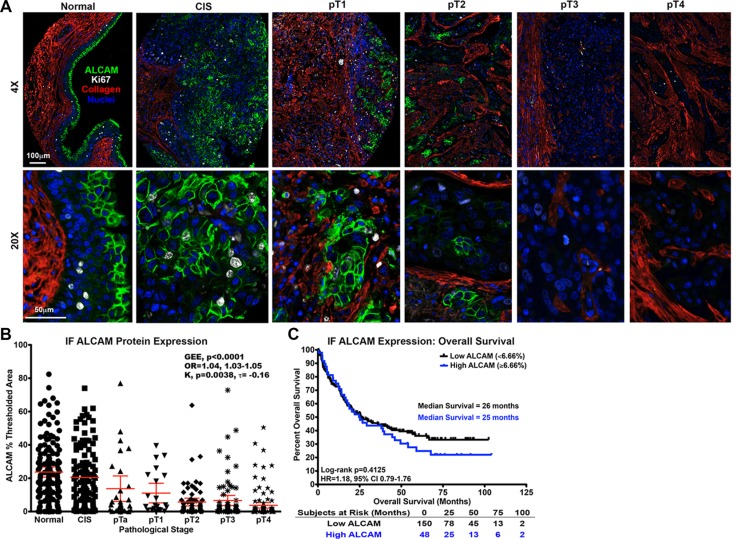
Correlation of intratumoral ALCAM protein expression with tumor stage and overall survival in bladder cancer Correlation of ALCAM protein expression by immunofluorescence (IF) in the BCa TMA cohort with tumor stage (**A**, **B**) and overall survival (**C**). (A) IF for ALCAM (green), Ki67 (white), Collagen (red), and Nuclei (blue) in normal bladder and BCa (CIS, pT1, pT2, pT3, pT4). Scale bars = 100 μm (low magnification) and 50μm (high magnification). (B) Correlation of core stage (*n* = 481) and ALCAM IF percent thresholded area with GEE ordinal logistic regression (OR, odds ratio; with 95% CI) and Kendall's (K) rank correlation (τ). Mean and 95% CI displayed. (C) Kaplan-Meier curves and Log-rank testfor overall survival (*n* = 198) with ALCAM IF expression dichotomized around the mean percent thresholded area of 6.66%. HR, Hazard Ratio. CI, Confidence Interval.

To determine if protein expression of ALCAM in BCa correlates with tumor stage and/or patient outcome, we performed immunofluorescence staining on tissue microarrays (TMAs) constructed of high-grade BCa specimens collected during cystectomy (Table 2) as described in the methods. The final readout for ALCAM was a continuous variable defined as the area within the region of interest (epithelium) that was above background (% thresholded area). In normal bladder, ALCAM protein expression was confined to the urothelium (Figure 2A, Normal). In non-invasive carcinoma *in situ*, the expansion of the urothelium led to an increase in ALCAM positive cells with no increase in signal intensity (Figure 2A, CIS). However, concomitant with the appearance of an invasive phenotype, ALCAM detection and fluorescence intensity diminished in the progression from pT1 to pT4 (Figure 2A).

**Table 2:** Bladder cancer TMA ALCAM expression cohort descriptors and frequencies

	*N*	Quartiles	Mean ± SD
Age (Years)	198	59, 67, 73	66 ± 11
ALCAM % Thresholded Area (IF)	198	0.33, 1.64, 6.50	6.56 ± 11.69
Follow-up (Months)	198	9.3, 25.4, 54.0	33.4 ± 27.3
Time to Death 3 Years (Months)	109	4.2, 12.6, 18.7	12.4 ± 9.0
Time to Death (Months)	130	5.5, 14.1, 25.5	18.4 ± 16.4
	**N**	**Percent**	**Frequency**
Gender	198		
Female		21.2%	42
Male		78.8%	156
Race	198		
White		93.4%	185
Black		4.5%	9
Other		2.0%	4
Death (Full Follow-up)	198		
0		34.3%	68
1		65.7%	130
Death (3 years)	198		
0		44.9%	89
1		55.1%	109
Pathological Tumor Stage	198		
pTa^Ψ^		0.5%	1
pTis^Ψ^		0.5%	1
pT1		10.6%	21
pT2a		17.2%	34
pT2b		18.7%	37
pT3a		21.2%	42
pT3b		15.7%	31
pT4a		15.2%	30
pT4b		0.5%	1
N Stage	198		
N0		71.2%	141
N1		10.6%	21
N2		18.2%	36
Core Stage	481		
Normal		29.3%	141
pTa		5.6%	27
pTis		24.3%	117
pT1		4.4%	21
pT2		14.8%	71
pT3		15.2%	73
pT4		6.4%	31

Description of the bladder cancer TMA patient cohort used for prognostic assessment of ALCAM expression by immunohistochemistry and immunofluorescence. There are 301 patients represented in the TMA with 657 cores total. Cores were classified by a pathologist as normal, pTis, pTa or “invasive” with each patient having multiple stages represented in the TMA. Only patients with invasive cores (*n* = 198) were utilized for overall survival analysis to prevent patients from being represented more than once. The mean of identically classified cores for each patient (*n* = 481) was calculated and used in the correlation of ALCAM expression with core stage. Quartiles (25%, Median, 75%) along with mean and standard deviation (SD) are given. Ψ, pathologist classified core as invasive even though the pathological staging was pTa and CIS.

Since each patient had multiple cores representing several pathological stages and, thus, had non-independent samples, the correlation of the mean ALCAM intensity score with pathological core stage was analyzed with generalized estimating equations (GEE) ordinal logistic regression and Kendall's rank correlation (K) (*n* = 481). Based on these analyses, ALCAM was significantly and inversely correlated with core stage, demonstrating a loss of ALCAM detection with advanced stage (K, τ = −0.16; *p* = 0.004; GEE OR, 1.04; 95% CI, 1.03–1.05; *p* < 0.0001; Figure 2B). In other words, there is a 4% increased odds of higher stage with every 1% decrease in ALCAM thresholded area. However, subsequent overall survival analysis performed using only invasive core values (*n* = 198) revealed that ALCAM expression failed to correlate with overall survival when percent thresholded area was dichotomized around the mean of 6.66% (Log-Rank, *P* = 0.413; HR, 1.18; 95% CI, 0.79–1.76; Figure 2C). Most importantly, ALCAM protein expression was not a significant predictor of overall survival when treated as a continuous covariate and adjusted for age, gender, tumor stage and lymph-node status by multivariable Cox regression analysis (Table 1 Bottom; adjusted HR, 1.00; 95% CI, 0.99–1.02; *p* = 0.843). These observations demonstrate that, in spite of a strong correlation between ALCAM protein detection and tumor stage, ALCAM expression fails to independently correlate with or predict patient outcome.

### ALCAM shedding

**Figure 3:**
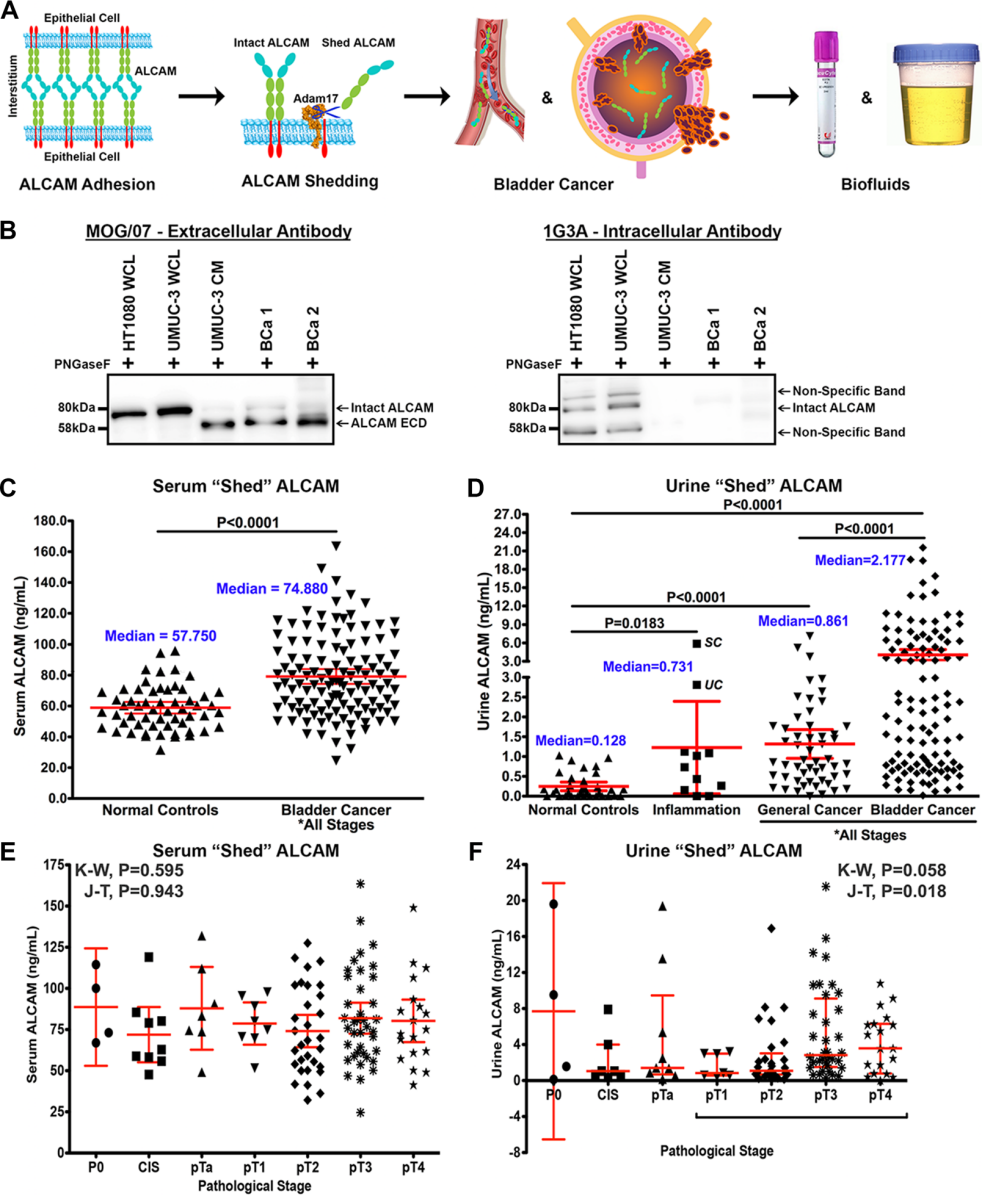
Detection of shed ALCAM in biofluids of patients with bladder cancer (**A**) The extracellular domain of ALCAM is cleaved from the cell surface by ADAM17 when tumor cells become invasive and can be detected in tumor-adjacent biofluids such as blood and urine. (**B**) ALCAM immunoblots of deglycosylated (PNGaseF) tumor cell lysates (WCL), conditioned media (CM) and urine (BCa 1 and BCa 2) probed with either antibodies to the extracellular domain (MOG/07) or intracellular domain (1G3A [23]) of ALCAM. Arrows indicate full-length (Intact ALCAM) and the cleaved ALCAM extracellular domain (ALCAM ECD). (**C**) ALCAM levels (ng/ml) in serum of normal controls compared to patients with BCa. (**D**) ALCAM levels (ng/ml) in urine of normal controls compared to patients with inflammatory conditions, cancers other than BCa, and BCa. Note segmented y-axis. UC, ulcerative colitis. SC, staghorn calculi. (**E**, **F**) Correlation of shed ALCAM in the serum (E) and urine (F) with pathological tumor stage in the VUMC shed ALCAM BCa cohort (*n* = 120). K-W, Kruskal-Wallis test. J-T, Jonckheere-Terpstra test for trend. Graphs display mean and 95% CI.

While the detection of ALCAM protein within the tumor tissue is reduced with disease progression, such a trend was not observed in gene expression. Since immunofluorescence staining for ALCAM was performed with an antibody against the extracellular domain, we suspected that the loss of ALCAM in BCa tissue was likely due to proteolytic shedding of the ectodomain [20, 21]. Consequently, we hypothesized that ALCAM shed by malignant urothelium should be detectable in adjacent biofluids such as blood and urine (Figure 3A).

We first wanted to determine if ALCAM is detectable in biofluid and, if so, verify that it is, indeed, shed ALCAM and not just intact ALCAM derived from cellular debri or exosomes. Not only was ALCAM detectable, immunoblots of urine from patients with bladder cancer as well as, from tumor cell lysates and conditioned media, reveal two pieces of evidence verifying biofluid ALCAM is actually proteolytically cleaved, shed ALCAM (Figure 3B). First, there is a downward shift in the size of ALCAM detected in conditioned media and urine compared to that detected in lysate (MOG/07; Figure 3B, Left). Second, the monoclonal antibody, 1G3A, that is against the intracellular domain of ALCAM can only detect ALCAM in the cell lysate, indicating that ALCAM in the tumor cell conditioned media and urine is lacking this intracellular domain (1G3A; Figure 3B, Right).

### Shed ALCAM levels in serum and urine

We next analyzed serum and urine ALCAM levels by ELISA from patients in four distinct biofluid cohorts including: 1) patients undergoing surgery but with no cancer (Normal Controls), 2) patients with inflammatory diseases (Inflammation), 3) patients with non-bladder malignancies (General Cancer) and 4) patients with high-grade bladder cancer (BCa). Analysis of serum ALCAM revealed that it was moderately elevated in BCa patients compared to Normal Controls (Figure 3C; 1.3-fold, *P* < 0.0001). In contrast, the level of ALCAM in urine from BCa patients was dramatically elevated when compared to Normal Controls (Figure 3D; 17.0-fold, *P* < 0.0001). Urinary ALCAM levels were also measured for patients with inflammatory diseases or other cancers to confirm that the significant elevation of urine ALCAM was specific to the presence of BCa. These non-BCa urines did show elevated levels of ALCAM when compared to normal controls (Figure 3D; 5.6 and 6.6-fold; *P* = 0.0183 and *P* < 0.0001) but still contained significantly less ALCAM than the BCa urines (Figure 3C; 3.0 and 2.5-fold; both *P* < 0.0001).

### Quality control

Quality control assays were performed to ensure that a commercially available ELISA test of ALCAM was sufficiently repeatable (i.e. reproducible within the same laboratory). Urinary ALCAM measurements were not significantly influenced by freeze-thaw (Supplementary Figure S1A), collection method (Supplementary Figure S1B; foley-derived vs. clean catch urine), or assay variation (Supplementary Figure S1C and S1D; 4–12% inter-assay variation).

### Shed ALCAM and univariable overall survival correlation

**Figure 4:**
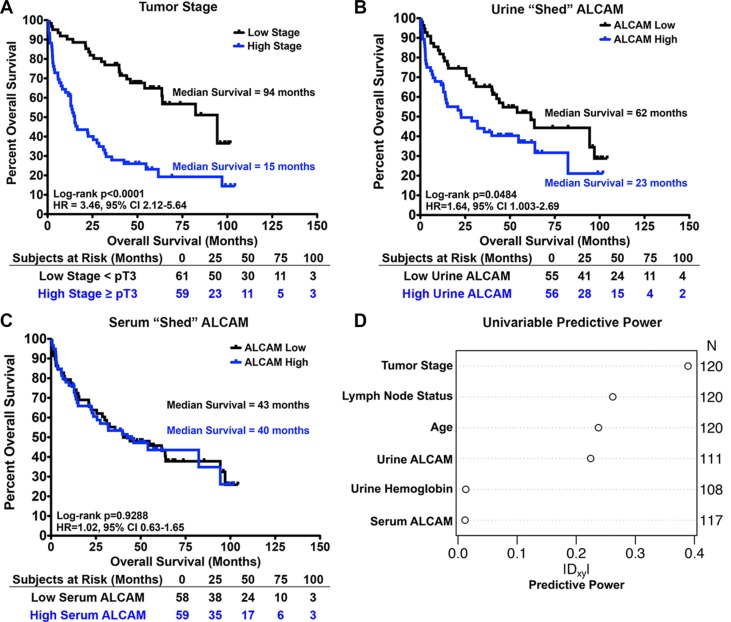
Univariable correlation of shed ALCAM with tumor stage and overall survival in bladder cancer Kaplan-Meier curves and Log-rank tests for analysis of overall survival with tumor stage (**A**), urine ALCAM (**B**), and serum ALCAM (**C**). (A) High stage ≥ pT3 and low stage < pT3. (B, C) Serum and urine ALCAM were dichotomized as high and low around the median (serum = 74.9 ng/ml and urine = 2.2 ng/ml). HR, Hazard Ratio. CI, Confidence Interval. (**D**) Univariable predictive power measured by Somers' Dxy rank correlation with 3-year OS allowing censoring for each predictor.

Since both serum and urine ALCAM concentrations were elevated in BCa, we set out to determine if either correlated with tumor stage and/or patient outcome using the VUMC cohort (Table 3, *n* = 120). Serum ALCAM levels did not show correlation with pathological tumor stage (Figure 3E; Kruskal-Wallis (K-W), *P* = 0.595; Jonckheere-Terpstra test for trend (J-T), *P* = 0.943). Urine ALCAM levels were not significantly different between tumor stages but did show a significant positive trend (Figure 3F; K-W, *P* = 0.058; J-T, *P* = 0.018), suggesting that ALCAM shedding increases with invasive progression. Next, Kaplan-Meier curves for overall survival were plotted for tumor stage, urine ALCAM and serum ALCAM (Figure 4A–4C). As expected, advanced tumor stage (≥ pT3, high stage) significantly correlated with decreased survival (Figure 4A; median overall survival (OS), 94 vs. 15 months; Log-Rank, *P* < 0.0001; HR, 3.46; 95% CI, 2.12–5.64). Urinary ALCAM dichotomized around the median of 2.18 ng/ml also significantly stratified patients into high and low risk of death (Figure 4B; median OS, 62 vs. 23 months; Log-Rank, *P* = 0.048; HR, 1.64; 95% CI, 1.003–2.69). However, serum ALCAM dichotomized around the median of 74.88 ng/ml did not show correlation with overall survival (Figure 4C; Log-Rank, *P* = 0.929; HR, 1.02; 95% CI, 0.63–1.65). In order to evaluate the potential predictive power or confounding effect of each variable in our multivariable regression analysis, we computed Somers' Dxy rank correlation between each variable and 3-year OS time considering censoring (univariable predictive power) [39]. Tumor stage, positive lymph-node status, age and urine ALCAM had relatively high correlation with survival time compared to urine hemoglobin and serum ALCAM (Figure 4D). Therefore, we chose to exclude serum ALCAM from subsequent multivariable analyses but retain urine hemoglobin as an *a priori* defined control for urine ALCAM since there is no other way to exclude the possibility that elevated urine ALCAM could be a result of hematuria rather than direct tumor shedding.

**Table 3:** Bladder cancer “shed” ALCAM cohort descriptors and frequencies

	VUMC (*n* = 120)			UTSW (*n* = 64)			Comparison		Combined Cohort (*n* = 184)		
	*N*	Quartiles	Mean ± SD	*N*	Quartiles	Mean ± SD	Test Statistic	Significance	*N*	Quartiles	Mean ± SD
Age (Years)	120	62, 69, 75	68 ± 10	63	63, 70, 77	69 ± 10	U = 3434^a^	0.311	183	63, 69, 75	68 ± 10
Serum ALCAM (ng/ml)	117	58.8, 74.9, 96.7	79.2 ± 26.3	29	53.6, 61.8, 68.6	69.1 ± 36.8	U = 1096^a^	0.003^*^	146	57.6, 70.7, 90.5	77.2 ± 28.8
Urine ALCAM (ng/ml)	111	0.78, 2.18, 6.18	4.05 ± 4.68	64	0.68, 1.35, 2.51	2.08 ± 2.24	U = 2755^a^	0.013^*^	175	0.73, 1.72, 4.24	3.33 ± 4.07
Urine Hemoglobin (μg/ml)	108	45, 341, 1088	1285 ± 2923	63	301, 503, 1055	1205 ± 2968	U = 2706^a^	0.025^*^	171	128, 448, 1088	1256 ± 2931
Follow-up (Months)	120	12.6, 38.1, 57.1	38.0 ± 57.1	59	5.0, 12.5, 29.3	18.3 ± 15.9	U = 2170^a^	< 0.001^*^	179	8.7, 26.0, 49.3	31.5 ± 27.3
Time to Death 3 Years (Months)	56	2.8, 11.1, 21.7	12.5 ± 10.4	20	1.7, 7.3, 17.1	9.5 ± 9.2	U = 455^a^	0.219	76	2.7, 9.3, 20.9	11.7 ± 10.1

	N	Percent	Frequency	N	Percent	Frequency	Test Statistic	Significance	N	Percent	Frequency
Gender	120			62			χ^2^_1_ = 3.23^b^	0.224	182		
Female		10.0%	(12)		9.7%	(6)				9.9%	(18)
Male		90.0%	(108)		90.3%	(56)				90.1%	(164)
Race	120			64			χ^2^_2_ = 8.32^b^	0.014^*^	184		
White		95.8%	(115)		89.1%	(57)				93.5%	(172)
Black		2.5%	(3)		4.7%	(3)				3.3%	(6)
Other		1.7%	(2)		6.3%	(4)				3.3%	(6)
Pathological Tumor Stage	120			63			χ^2^_8_ = 5.66^b^	0.586	183		
pT0		4.2%	(5)		7.9%	(5)				5.5%	(10)
pTa		6.7%	(8)		1.6%	(1)				4.9%	(9)
pTis		7.5%	(9)		7.9%	(5)				7.7%	(14)
pT1		6.7%	(8)		9.5%	(6)				7.7%	(14)
pT2a		16.7%	(20)		19.0%	(12)				17.5%	(32)
pT2b		9.2%	(11)		15.9%	(10)				11.5%	(21)
pT3a		25.8%	(31)		17.5%	(11)				23.0%	(42)
pT3b		7.5%	(9)		7.9%	(5)				7.7%	(14)
pT4a		15.8%	(19)		12.7%	(8)				14.8%	(27)
N Stage	120			60			χ^2^_3_ = 5.85^b^	0.088	180		
N0		75.0%	(90)		73.3%	(44)				74.4%	(134)
N1		5.0%	(6)		10.0%	(6)				6.7%	(12)
N2		20.0%	(24)		13.3%	(8)				17.8%	(32)
N3		0.0%	()		3.3%	(2)				1.1%	(2)
Positive Lymph-node Status	120			60			χ^2^_1_ = 0.06^c^	0.809	180		
0		75.0%	(90)		73.3%	(44)				74.4%	(134)
1		25.0%	(30)		26.7%	(16)				25.6%	(46)
Group	120			0					120		
NMIBC		25.0%	(30)							25.0%	(30)
OCMIBC		24.2%	(29)							24.2%	(29)
EVMIBC		25.8%	(31)							25.8%	(31)
LN+		25.0%	(30)							25.0%	(30)
Death (Full Follow-up)	120			58			χ^2^_1_ = 9.53^c^	0.002^*^	178		
0		40.8%	(49)		65.5%	(38)				48.9%	(87)
1		59.2%	(71)		34.5%	(20)				51.1%	(91)
Death (3 years)	120			58			χ^2^_1_ = 2.37^c^	0.147	178		
0		53.3%	(64)		65.5%	(38)				57.3%	(102)
1		46.7%	(56)		34.5%	(20)				42.7%	(76)

Description and comparison of the retrospective bladder cancer patient cohorts used for prognostic assessment of shed ALCAM in serum and urine collected at VUMC and UTSW. a = Mann-Whitney U, b = Fisher's Exact, c = Pearson Chi Square, * exact 2-tailed significance detected, Quartiles = 25%, Median, 75%, NMIBC = non-muscle invasive BCa, OCMIBC = organ-confined muscle invasive BCa, EVMIBC = extravesical muscle invasive BCa, LN+ = lymph-node positive muscle invasive BCa

### Urinary ALCAM and multivariable analysis for prediction of 3-year overall survival

**Figure 5:**
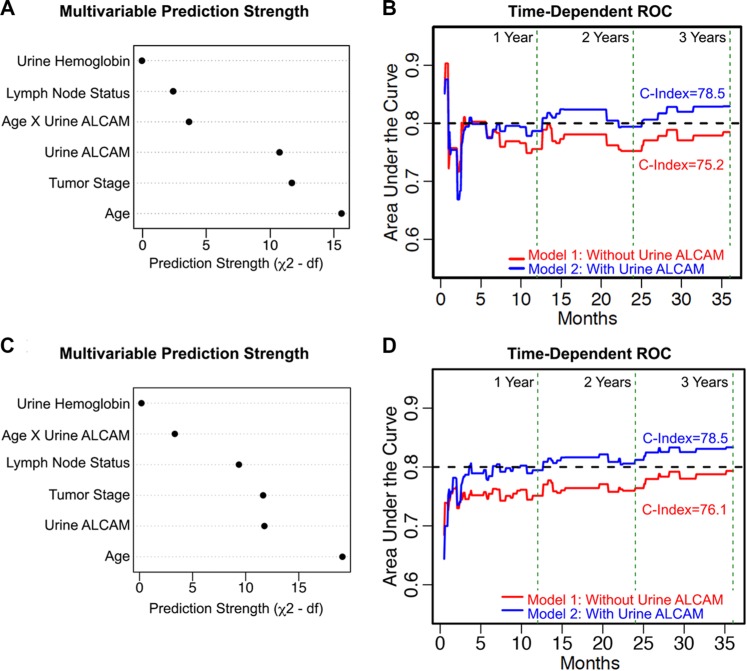
Multivariable prediction of 3-year overall survival in bladder cancer (**A**, **C**) Adjusted Chi-square statistics for all the variables in Model 2 for the VUMC cohort alone (A) and the combined VUMC and UTSW cohort (C). (**B**, **D**) Time-dependent receiver operating characteristic (ROC) curves for Model 1 (red) compared with Model 2 (blue) for the VUMC cohort alone (B) and the combined cohort (D). Concordance indices (C-Index). Vertical green dotted lines mark 12, 24 and 36 months. Black dotted line marks 0.80 (80%) concordance.

To determine if urine ALCAM was an independent predictor of OS, multivariable Cox regression analyses were performed on this same retrospective biofluid cohort (Table 3, VUMC, *n* = 120). Race and gender were excluded from all multivariable analyses since the VUMC cohort is 95.8% white and 90.0% male. As stated previously, urine hemoglobin was retained in further analyses since there was concern that hematuria would be a confounder for urine ALCAM. Therefore, the baseline model (Model 1) that was used to assess the benefit of adding urine ALCAM to predict 3-year OS included age, tumor stage, positive lymph-node status, and urine hemoglobin (Table 4). As expected, age, tumor stage, and positive lymph-node status were all independent predictors of 3-year OS (Table 4; Model 1; *P* < 0.001, *P* = 0.006, *P* = 0.009). Importantly, after adjusting for these parameters and urinary hemoglobin, urine ALCAM was also a significant independent predictor of 3-year OS (Table 4; Model 2; *P* = 0.002). Of note, the multivariable prediction strength of urine ALCAM nearly matches that of tumor stage based on adjusted partial likelihood ratio Chi-square statistics (Figure 5A). By setting the age to 69 and the urine ALCAM to 2.06, we were able to calculate the interaction adjusted hazard ratios and confidence intervals for each of the parameters in the model; whereby, patients are at 1.5 times greater risk of death within 3 years following cystectomy if their urine ALCAM level is high (6.0 ng/ml) compared to low (0.75 ng/ml) (95% CI, 1.03–2.06; *P* = 0.002). Moreover, this effect is significantly modified by age (Table 4; Model 2, Urine ALCAM X Age, *P* = 0.031). To visualize this interaction, the adjusted effects of urine ALCAM were plotted at different age groups for patients with tumor stage 4, negative lymph-node status, and 341 ng/ml of urine hemoglobin (Supplementary Figure S2A). Importantly, internal validation analyses revealed that there was no significant over-fitting of the models (Supplementary Figure S3A).

**Table 4:** Assessment of urinary “shed” ALCAM as a predictor in a multivariable Cox regression analysis of 3-year overall survival in the VUMC bladder cancer cohort

	Variable	Hazard Ratio	95.0% CI	Significance	Bootstrap Significance
Model 1	Age (Years)	1.06^a^	1.03–1.10	< 0.001	0.001
	Tumor Stage	1.28^a^	1.07–1.52	0.006	0.008
	Lymph-node Positive	2.62^a^	1.27–5.38	0.009	0.016
	Urine Hemoglobin (μg/ml)	1.00^a^	0.99–1.00	0.116	0.189
	C-Index	75.2%	68.8–81.5		73.5^Ψ^
Model 2	Age (Years)	1.74^b^	1.05–2.87	< 0.001	0.001
	Tumor Stage	1.98^c^	1.36–2.89	< 0.001	0.002
	Lymph-node Positive	2.03	0.96–4.30	0.065	0.034
	Urine Hemoglobin (μg/ml)	1.07^d^	0.93–1.23	0.329	0.729
	Urine ALCAM (ng/ml)	1.46^e^	1.03–2.06	0.002	0.005
	Urine ALCAM X Age^*^			0.031	0.023
	C-Index	78.5%	72.4–84.6		76.0^Ψ^
Model 3	Age (Years)	1.06	1.02–1.11	0.004	0.020
	Tumor Stage	1.37	1.06–1.76	0.015	0.022
	Lymph-node Positive	1.60	0.64–4.02	0.314	0.343
	Urine Hemoglobin (μg/ml)	1.00	0.99–1.00	0.435	0.529
	Urine Creatinine (mg/dl)	1.00	0.99–1.00	0.875	0.889
	Urine ALCAM (ng/ml)	1.10	1.02–1.19	0.011	0.018
	BCG Treatment	0.62	0.29–1.36	0.233	0.310
	CIS Present	1.79	0.86–3.72	0.119	0.129
	# Lymph-nodes Removed	1.00	0.95–1.04	0.918	0.931
	Lymphovascular Invasion	1.45	0.53–3.98	0.467	0.549
	Adjuvant Chemotherapy	0.77	0.30–1.96	0.581	0.634

Assessment of urinary shed ALCAM as a predictor of 3-year overall survival by multivariable Cox regression analysis in the VUMC cohort. Hazard Ratio is the adjusted hazards ratio. CI, confidence interval. BCG, Bacillus Calmette-Guérin. CIS, carcinoma *in situ*. Bootstrap significance is two-tailed with 1000 iterations and a Mersenne twister of 2,000,000. Hazard ratio is the adjusted hazard ratio for

a every 1 unit increase,

b urine ALCAM at 2.06 ng/ml, but 13 years older,

c 2 tumor stage increase,

d 1.03 μg/ml higher urine hemoglobin, and

e 69 year old with a 5.03 ng/ml increase in urine ALCAM.

* Interaction term. C-Index, Harrell's Concordance Index [41].

Ψ internal validation of the C-Index, confidence interval calculations not available.

The ability of urine ALCAM to improve the prediction of 3-year OS (Model 2) when compared to standard predictors alone (Model 1) was assessed by graphing reclassification plots and time-dependent receiver operating characteristics curves (ROC) based on multivariable Cox regression analyses. Reclassification plots, where the predicted risk without urine ALCAM (Model 1) is plotted against the predicted risk with urine ALCAM (Model 2), showed that the addition of urine ALCAM was effective at reclassifying patients with high and low risk of death; whereby, event points (open circles) were mainly shifted above the diagonal and non-event points (black circles) were mainly shifted below the diagonal, which is in agreement with what is expected if there is improvement in risk prediction (Supplementary Figure S2B). Additionally, the computed continuous ½ net reclassification index (NRI) [40] at 1 year post-surgery is 31.5% (95% CI, 0.00–0.52; *P* = 0.05), which indicates that 31.5% of patients see an improvement in risk prediction with the addition of urine ALCAM. Furthermore, the time dependent ROC curves show that over the 3 years of follow-up, there was a 3–5% increase in area under the curve (AUC) with the addition of urine ALCAM (Figure 5B). However, this is only true after the first 6 months of follow-up where it is speculated that, prior to this time, patient deaths are due to post-surgical complications and lack of recovery. There was also a 3.3% increase in Harrell's Concordance Index (C-Index) [41] (Table 4; 75.2% vs. 78.5%). Additionally, after internal Bootstrap validation to correct for any over-fitting, the inclusion of urine ALCAM still showed a clinically meaningful improvement of 2.5% in the C-Index (Table 4; 73.5% vs. 76.0%). Importantly, urine ALCAM still remained an independent predictor of OS after accounting for additional clinical features including treatment with BCG, presence of carcinoma *in situ* (CIS), extent of lymph-node dissection, lymphovascular invasion, urine creatinine, and adjuvant chemotherapy (HR, 1.10; 95% CI, 1.02–1.19; *P* = 0.011; Table 4, Model 3).

In summary, the addition of urine ALCAM is an independent prognostic indicator and improves the prediction of post-cystectomy, 3-year overall survival of patients with BCa to a degree that is clinically relevant using the VUMC cohort.

### Validation

In order to validate the VUMC cohort results, a similar retrospective BCa cohort containing matched serum and urine collected at time of cystectomy was obtained from University of Texas Southwestern (Table 3; UTSW, *n* = 64). The UTSW and the VUMC cohorts were significantly different on several parameters including urine and serum ALCAM, urine hemoglobin, follow-up time, and race (Table 3). However, urine ALCAM was still significantly elevated in the UTSW cohort compared to normal controls (10.6-fold, *p* < 0.0001). The follow-up for the UTSW cohort was shorter than the VUMC cohort (Table 3; median 12.5 vs. 38.1 months) but the 3-year overall survival of the two cohorts were similar (Table 3). Since there were not enough events in the UTSW cohort for multivariable analysis (events = 20), we chose to combine the VUMC and UTSW cohorts to strengthen the generalizability of the prediction model (Table 3; events = 76).

Again, in the combined cohort, age, tumor stage, and positive lymph-node status were all independent predictors of 3-year OS (Table 5; Model 1; *P* < 0.0001, *P* = 0.003, *P* < 0.001). The interaction between urine ALCAM and age remained significant (Supplementary Figure S2C and Table 5; Model 2, Urine ALCAM X Age, *P* = 0.038). Importantly, urine ALCAM remained a significant independent predictor of 3-year OS after adjusting for baseline parameters and the age interaction (Table 5; Model 2; adjusted HR, 1.27; 95% CI, 1.05–1.52, *P* = 0.001). Interaction adjusted hazard ratios and confidence intervals were calculated by setting the age to 69 and the urine ALCAM to 1.61 in the interaction term and revealed that patients have a 30% increased risk of death within 3 years following surgery if their urine ALCAM levels are high (4.05 ng/ml) compared to low (0.69 ng/ml) (95% CI 1.05–1.52, *P* = 0.001). Urine ALCAM still had similar prediction strength as tumor stage based on adjusted partial likelihood ratio Chi-square statistics (Figure 5C). Likewise, internal bootstrap validation revealed no significant over-fitting of model 1 and model 2 for the combined cohort (Supplementary Figure S3B).

**Table 5:** Assessment of urinary “shed” ALCAM as a predictor in a multivariable Cox regression analysis of 3-year overall survival in the combined VUMC and UTSW bladder cancer cohort

	Variable	Hazard Ratio	95.0% CI	Significance	Bootstrap Significance
Model 1	Age (Years)	1.05^a^	1.03–1.08	< 0.0001	0.001
	Tumor Stage	1.25^a^	1.08–1.45	0.003	0.001
	Lymph-node Positive	2.95^a^	1.64–5.28	< 0.001	0.002
	Urine Hemoglobin (μg/ml)	1.00^a^	0.99–1.00	0.073	0.072
	C-Index	76.1%	70.7–81.4		75.2^Ψ^
Model 2	Age (Years)	1.58^b^	1.08–2.34	< 0.0001	0.001
	Tumor Stage	1.75^c^	1.28–2.37	< 0.001	0.002
	Lymph-node Positive	2.69	1.47–4.92	0.001	0.002
	Urine Hemoglobin (μg/ml)	1.04^d^	0.97–1.12	0.278	0.463
	Urine ALCAM (ng/ml)	1.27^e^	1.05–1.52	0.001	0.005
	Urine ALCAM X Age^*^			0.038	0.014
	C-Index	78.5%	73.4–83.7		76.8^Ψ^
Multiple Imputation	Age (Years)	1.52^f^	1.07–2.17	0.0002	
	Tumor Stage	2.34^g^	1.48–3.69	0.0003	
	Lymph-node (N Stage)	1.66^h^	1.24–2.21	0.0006	
	Urine Hemoglobin (μg/ml)	1.04^i^	0.95–1.13	0.408	
	Urine ALCAM (ng/ml)	1.22^j^	1.00–1.49	0.018	
	Urine ALCAM X Age^*^			0.078	

Assessment of urinary shed ALCAM as a predictor of 3-year overall survival by multivariable Cox regression analysis in the combined VUMC and UTSW cohort. CI, confidence interval. Bootstrap significance is two-tailed with 1000 iterations and a Mersenne twister of 2,000,000. Hazard ratio is the adjusted hazard ratio for

a every 1 unit increase,

b urine ALCAM at 1.61 ng/ml, but 8 years older,

c 2 tumor stage increase,

d 0.90 μg/ml higher urine hemoglobin, and

e 69 year old with a 3.36 ng/ml increase in urine ALCAM.

* Interaction term. C-Index, Harrell's Concordance Index [41].

Ψ internal validation of the C-Index, confidence interval calculations not available. Multivariable Cox regression analysis with Model 2 in the combined VUMC and UTSW cohort after replacement of missing data by multiple imputation via Bayesian Bootstrap Predictive Mean Matching (PMM) [51, 52]. Hazard ratio is the adjusted hazard ratio for

f urine ALCAM at 1.72 ng/ml, but 12 years older,

g 3 tumor stage increase,

h 1 lymph-node stage (N Stage, 0–3) increase,

i 0.96 μg/ml higher urine hemoglobin, and

j 69 year old with a 3.50 ng/ml increase in urine ALCAM.

Furthermore, similar to the VUMC cohort alone, the addition of urine ALCAM in the multivariable model tended to increase the predicted risk for event patients and decrease the predicted risk for non-event patients, thus improving the classification (Supplementary Figure S2D). In addition, the combined cohort time-dependent ROC curves show that over the 3 years of follow-up, there was a 4–5% increase in AUC with the addition of urine ALCAM (Figure 5D). Most importantly, the addition of urine ALCAM in the multi-institutional cohort still showed a clinically meaningful improvement of 2.4% in the C-Index and, after internal validation, this increase was still 1.6% (Figure 5D and Table 5; C-Index, 76.1 vs. 78.5; bootstrap validation, 75.2 vs. 76.8).

As a final analysis, we performed multiple imputation using Bayesian Bootstrap Predictive Mean Matching (PMM) on the combined cohort as a method to estimate values for missing data. After multiple imputation, urine ALCAM remained an independent predictor of 3-year OS (Table 5; adjusted HR, 1.22; 95% CI, 1.00–1.49, *P* = 0.018).

**Figure 6:**
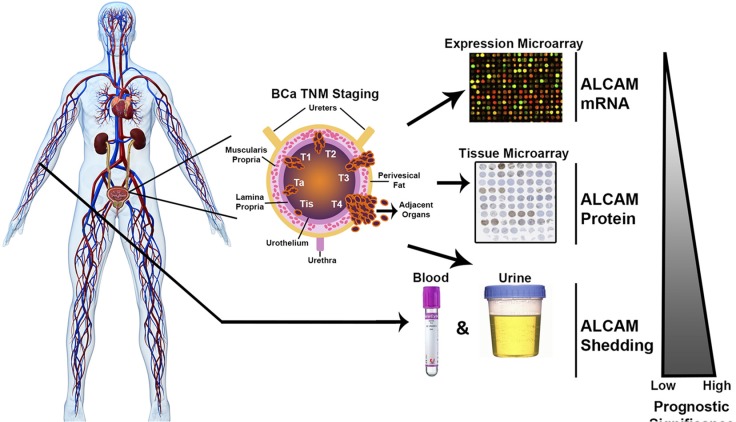
Summary of the multi-level approach for analysis of ALCAM in bladder cancer In the current study, we evaluate the prognostic significance of ALCAM mRNA, protein, and shedding in regard to overall survival in bladder cancer. The urothelium expresses an abundance of ALCAM. ALCAM is then cleaved from the cell surface by the protease ADAM17 during invasive progression of cancer. ALCAM shed from malignant urothelium should be detectable and elevated in adjacent fluids such as serum and urine. Our data supports the hypothesis that ALCAM shedding, which is a functional read-out of tumor cell migration and thus, invasion and metastasis, has greater prognostic value than its expression.

In summary, we show that although the histological detection of ALCAM within the tumor tissue correlates strongly with tumor stage in BCa (Figure 2), it does not appear to be prognostic of overall survival. In contrast, urine ALCAM correlates with tumor stage and is a significant independent predictor of 3-year overall survival for patients after cystectomy. All results are summarized in Figure 6, which emphasizes the discordance between correlation with stage and correlation with outcome.

## DISCUSSION

Although intervention can be curative for BCa patients, 50% of patients experience metastatic recurrence within two years following cystectomy [5]. Patient outcome could improve if: 1) patients with a low-grade, non-invasive BCa who are at risk of rapid progression could be identified for earlier radical surgical intervention and 2) patients with high-grade and/or invasive BCa at risk of metastatic recurrence could be identified for more aggressive intervention such as neo-adjuvant or adjuvant chemotherapy. Prognostic biomarkers can aid in identification of such high-risk patients. Urinary biomarkers have additional clinical value in that they provide a longitudinal and non-invasive means to monitor tumor progression, recurrence and treatment response. Molecular products that are mechanistically involved in or directly result from cell motility make particularly attractive biomarkers because tumor cell migration is a central driver of malignant progression and metastatic dissemination [42]. These molecular motility markers could predict or detect disease progression before overt clinical manifestation. ALCAM forms adhesive interactions between neighboring epithelial cells but cohesion is disrupted by membrane proximal, proteolytic cleavage and release of the ALCAM ectodomain from mobile tumor cells [20, 21]. Therefore, ALCAM shedding is not specific to bladder cancer. Rather, the shed extracellular domain of ALCAM is a marker of invasive and metastatic disease and, thus, has the potential to be a clinically relevant prognostic biomarker in many epithelial cancers.

In agreement with our results, two recent publications looking at the correlation of ALCAM tissue expression with stage and outcome in breast cancer also reveal a loss in detectable levels of ALCAM by immunohistochemistry as tumors progress [43, 44]. This is consistent with our hypothesis that the loss of detection is due to increases in ALCAM shedding as tumors become invasive. Indeed, another group has shown in a diagnostic study that patients with breast cancer have elevated serum levels of ALCAM [45]. However, it has yet to be demonstrated that ALCAM shedding correlates with stage or outcome in breast cancer. We previously demonstrated in colorectal cancer that reduced detection of the ALCAM extracellular domain in tumor tissue is due to ALCAM shedding which, in turn, corresponds with poor patient outcome [23]. Those observations suggested that detection of shed ALCAM in adjacent biofluids could predict disease progression. This hypothesis was tested in our evaluation of urinary ALCAM from BCa patients. Indeed, there is a significant loss of intra-tumoral ALCAM during invasive transformation (Figure 2), while urinary ALCAM levels rise and correlate with poor outcome (Figures 3 and 4) (Figure 6). Further statistical interrogation provides evidence that urinary ALCAM is a significant independent predictor of overall survival after adjusting for age, tumor stage, positive lymph-node status, and urinary hemoglobin (Table 4) and improves accuracy of prediction (i.e. discrimination) by 3.3% (Figure 5). Furthermore, this observation was validated in a combined multi-institutional cohort (Figure 5 and Table 5).

Although our main hypothesis is in regards to prognosis, it is interesting to note that in the current study, serum ALCAM has a diagnostic accuracy, an area under the receiver operating characteristics curve, of 0.75 (*p* = 0.002, 95%CI 0.64–0.85) and urine ALCAM a 0.90 (*p* < 0.0001, 95%CI 0.85–0.94) in distinguishing all-stage bladder from normal and inflammatory controls combined. As a comparison, cystoscopy and cytology together are 80–99% accurate at diagnosing BCa and are more than commonplace in the clinic. Therefore, it is unlikely that shed ALCAM would provide any added diagnostic benefit in advanced, muscle-invasive bladder cancer. It would, however, be interesting to evaluate the diagnostic benefit of adding urine ALCAM to cytology in early-stage disease where cytology is much less accurate.

Our initial analysis was restricted to retrospective cohort studies based on the need for long-term follow-up in prognostication. While the retrospective nature of this study poses limitations in regards to confidence in extrapolation to larger populations due to biases such as lack of racial and gender diversity, incomplete recurrence data, and confounding associated with reporting, sample collection, clinicians, and/or institutional practices, we have removed some potential biases by combining two independent cohorts collected at VUMC and UTSW. Furthermore, we have applied rigorous bootstrap validation methods and a team of biostatisticians has independently validated all statistical analyses. Interestingly, in a small subset analysis of the VUMC retrospective cohort (*n* = 40), urine ALCAM was an even stronger predictor of metastatic recurrence (HR = 10.4), which should be expected for a molecule that is indicative of invasive disease. Unfortunately, recurrence data and/or disease specific survival was not available for a majority of the patients. Larger multi-institution and multi-country prospective cohort studies are ongoing to validate the prognostic utility of urinary ALCAM in BCa and will require 3 years of enrollment and an additional 3–5 years of follow-up. These prospective studies encompass repeated collection of biofluids and tissues over the course of progression, surgery, and (neo)adjuvant treatment, and will allow the tracking of cancer-specific outcome measures such as progression-free survival, treatment response, metastasis, and disease-specific survival.

The correlation between ALCAM shedding and patient outcome suggests that this process contributes to disease progression. Thus, therapeutic targeting of this process could limit disease progression and improve patient outcome. The promiscuity and critical roles of the sheddase, ADAM17, limit the utility of targeting the protease directly. However, the proteolytic fragments released by the shedding event are hypothesized to convey their own biological activity. Studies investigating how these fragments alter the tumor phenotype and how that mechanism can be targeted for intervention are currently ongoing.

In summary, we provide evidence that shed ALCAM is an independent prognostic biomarker for overall survival in BCa. Our findings also suggest potential utility of shed ALCAM in longitudinal, post-diagnostic surveillance and monitoring of treatment response. Detection of shed ALCAM in tumor-adjacent fluids makes it a promising non-invasive and cost-effective biomarker in BCa as well as other cancers with tumor-associated biofluids. We further speculate that, although urine ALCAM is the predictor in non-metastatic BCa, ALCAM shed into the blood will have prognostic relevance in patients with metastatic disease. Furthermore, since ALCAM contributes mechanistically to cell migration and metastasis, our findings provide evidence that the molecular status of a migratory mechanism can report on the clinical risk of disease progression. Finally, findings from this study suggest that focusing on protein function rather than expression alone has the potential to aid in biomarker discovery, development and implementation.

## MATERIALS AND METHODS

### Specimen collection

All specimen collections were approved by the Vanderbilt (VUMC) and University of Texas Southwestern (UTSW) Institutional Review Boards (IRB) and patient confidentiality was protected according to the U.S. Health Insurance Portability and Accountability Act (HIPAA). All fluids were stored at −80°C. Tissues were processed as standard diagnostic blocks and stored in the VUMC tissue library.

### Study populations

### ALCAM mRNA cohort

NCBI Gene Expression Omnibus (GEO, GSE31684) [32] was used to analyze ALCAM mRNA expression from excised cystectomy tumor tissue (probes 201951_at and 201952_at) in BCa and included 93 patients, representing stages pTa to pT4, who underwent radical cystectomy at Memorial Sloan-Kettering Cancer Center between 1993 and 2004. Median age of patients was 69.1 years, 73% were male, median follow-up was 32 months, and incidence of death was 70%. In addition, 3 other BCa GEO datasets were utilized to compare ALCAM mRNA expression in non-muscle invasive to muscle invasive disease (GSE48276, GSE13507, GSE3167) [29–31].

### ALCAM expression bladder cancer TMA cohort

Histological analysis of ALCAM protein expression was performed on a tissue microarray (TMA) from a retrospective cohort of patients undergoing radical cystectomy at VUMC from 2000–2010 for high-grade bladder cancer (301 patients, 657 total cores). The TMA was constructed from formalin-fixed, paraffin-embedded cystectomy diagnostic tissue blocks. Each patient contributed between 1 and 6 cores to the array, with matched core designations of adjacent normal, superficial (pTa and pTis/Cis) and invasive (≥ pT1). Immunofluorescence staining for ALCAM was performed and correlation with overall survival was analyzed for those patients with a designated “invasive” core (*n* = 198). Correlation of ALCAM expression with core pathology stage was performed on all unique cores (*n* = 481) as described in statistical methods. The mean immunofluorescence calculation was used when a core designation was represented more than once for a single patient, such as multiple normal cores, which is why there were only 481 unique cores of the 651 total cores.

### Shed ALCAM bladder cancer cohorts (VUMC)

The analysis of shed ALCAM in serum and urine, at time of surgery, was performed on a retrospective cohort of patients with high-grade bladder cancer undergoing radical cystectomy at VUMC from 2001–2006, which included pathological stages from pT0 to pT4 and excluded patients who had received neoadjuvant chemotherapy (*n* = 120).

### Urine control cohorts

The following age-matched, control clean-catch or catheter-derived urine specimens were collected at VUMC by the Cooperative Human Tissue Network: 1) Non-cancer control urines (Normal Controls) from patients with no history or current diagnosis of cancer undergoing non-urologic surgeries including cardiac bypass, gastric bypass, thyroidectomy, esophagomyotomy, knee replacement, and hernia repair, 2) Inflammation control urines (Inflammation) from patients with rectovaginal fistula, colorectal enteritis and ulceration, gallbladder polyploid cholesterolosis, endometriosis, atherosclerosis, ulcerative colitis, uterine fibroids, urethral stricture, and staghorn calculus, 3) Non-urologic cancer control urines (General Cancer) from patients with prostate, pancreatic, neuroendocrine, renal, and colorectal cancers.

### Non-cancer serum control cohort

Serum from age-matched, non-cancer patients was collected from discarded vitamin D clinical tests in the Vanderbilt Clinical Chemistry laboratory.

### Shed ALCAM bladder cancer cohort (UTSW)

For validation of urine ALCAM as a prognostic biomarker, our VUMC cohort was combined with a randomly selected retrospective cohort of patients with high-grade bladder cancer undergoing radical cystectomy at UTSW from 2005–2013, which included pathological stages from pT0 to pT4 (*n* = 64). Biofluids were collected at time of surgery.

### Immunofluorescence

Immunofluorescence (IF) was performed on the tissue microarray described above. Sections (5μm) were deparaffinized and rehydrated. Antigen retrieval was performed by pressure cooker in citrate buffer (pH 6.0) and sections blocked in 20% Aquablock (East Coast Biologics) plus 0.05% Tween-20. IF was performed with primary antibodies mouse anti-ALCAM (MOG/07; 1:100; Novocastra^TM^, Leica Biosystems), rabbit anti-Ki67 (Clone SP6; 1:500; Thermo Scientific), and Hoechst 33342 as well as, secondary antibodies Alexa-546 goat anti-rabbit and Alexa-647 goat anti-mouse (1:500; LifeTechnologies). Collagen was stained with Alexa 488-conjugated CNA35 (gift from Erin Rericha, Vanderbilt) [46, 47]. IF slides were mounted in ProLong Gold Antifade reagent (Invitrogen). Fluorescence intensity and thresholded area were quantified in the epithelium in each TMA core with an Image J-based batch macro. Collagen staining was used to distinguish between the epithelial, stromal and muscular compartments. Hoechst was used to define the nuclear compartment while Ki67 marked proliferating cells. Percent thresholded area of ALCAM was subsequently used for analysis.

### Urine ALCAM normalization

In order to assess the influence of hydration, proteinuria, and hematuria on urinary ALCAM levels, we initially aimed to include all these parameters in the multivariable models. Urinary total protein (Thermo Scientific, BCA, Cat# 23227), urinary creatinine (Enzo Life Sciences, Cat# 937–001), urinary specific gravity (Siemens Medical Solutions Diagnostics, Multistix^®^ 8 SG, Cat# 2164) and urinary hemoglobin (Sigma-Aldrich, Drabkin's Reagent, Cat# D5941) were all analyzed in a random subset of the VUMC cohort specimens. Unfortunately, the presence of urea made BCA analysis for total protein in urine unreliable and the limited dynamic range of specific gravity did not provide sufficient means to normalize. Urine creatinine neither added predictive value to the model nor altered the strength of urine ALCAM to predict overall survival. Therefore, only urine hemoglobin was considered in the final prediction model and analyzed in both the VUMC and UTSW cohorts.

### ALCAM immunoblotting

Immunoblotting for shed ALCAM was performed on urine from two patients with bladder cancer, the whole cell lysates from the fibrosarcoma cell line HT1080 and the bladder cancer cell line UMUC-3 as well as, UMUC-3 24-hour serum-free conditioned media. Urine and conditioned media were first precipitated with ice-cold acetone (1:4) at −20°C overnight, pelleted at 15,000 rpm for 15 minutes at 4°C, supernatant decanted and protein pellet air-dried for 15 minutes at room temperature. The protein pellet was then resuspended in lysis buffer (1.0% TritonX-100 in PBS) and sonicated at 37°C for 15 minutes. Next, all samples were deglycosylated using a PNGaseF kit according to the manufacturer's instructions (P0704s; New England Biolabs). Deglycosylated samples were then run on two identical 12% polyacrylamide gels for 30 minutes at 80 volts then 1.5 hours at 120 volts, transferred to a methanol-activated PVDF membrane in transfer buffer (25mM Tris, 192mM glycine, 20% methanol) for 2 hours at 100 volts, and blocked overnight with 5% milk. One blot was probed with a mouse monoclonal antibody against the extracellular domain (MOG/07; 1:1000; Abcam) and the other with our previously characterized in-house mouse monoclonal antibody against the intracellular domain (1G3A; 1:2000) [23] overnight at 4°C and then incubated with HRP-conjugated goat anti-mouse antibody (1:2500; Abcam) for 1 hour at room temperature. Blots were developed with West Fempta Enhanced Chemiluminescence reagent for 5 minutes and photons read for a total of 15 minutes in a digital light box (G:BOX; Syngene).

### ALCAM enzyme-linked immunosorbent assay

Serum and urine were analyzed by ALCAM ELISA according to the manufacturer's protocol (R&D Systems). All samples were analyzed in duplicate at dilutions (Urine: 4–8 fold; Serum: 50–80 fold) that matched the dynamic range of the assay (0.05–4.00 ng/ml).

### Statistical analysis

Statistical analyses were performed at a two-tailed significance of 0.05. Descriptive statistics such as mean and 95% confidence interval (CI) for ALCAM mRNA expression among different cohorts were graphed. Kruskal-Wallis and/or Wilcoxon rank-sum (Mann-Whitney U) tests were performed for comparing ALCAM mRNA level and ALCAM biofluid concentrations between independent groups such as different stages or cohorts. Jonckheere-Terpstra tests for non-independent groups were also used to assess trends of mRNA levels, protein levels or shed ALCAM concentrations with increasing tumor stage. To evaluate the association of ALCAM protein expression with core stage in the BCa TMA cohort, generalized estimating equations (GEE) ordinal logistic regression was used in order to account for the representation of multiple core stages for each patient (non-independent samples). Kendall's τ rank correlation was also calculated. Kaplan-Meier curves and log-rank tests were utilized for univariable survival analysis.

To assess the value of urine ALCAM as a biomarker, multivariable Cox regression analyses were performed using the VUMC and UTSW cohort data to predict overall survival (OS) of bladder cancer patients after cystectomy. OS time was defined as time from the date of cystectomy to date of death or last follow-up and was restricted to 3 years. The multivariable models were determined *a priori* based on each covariate's potential to confound or modify the association between shed ALCAM and survival as well as data availability. Urine hemoglobin was included in the model to adjust for bleeding in the urine (hematuria) and account for any contamination of urine with serum ALCAM. In the models, tumor stage, age, hemoglobin and urine ALCAM were modeled as continuous variables and lymph-node status as a binary variable. An interaction term between age and urine ALCAM was also included when the urine ALCAM was in the model. The models were internally validated using .632+ [48] bootstrapping and calibration accuracy for 2- and 3-year survival was also estimated using bootstrapping. The model results were also compared with those fitted using multiple imputed data. Time-dependent receiver operating characteristics (ROC) curves [49], Harrell's Concordance Index [41] and predicted risk scores were compared between models with and without urine ALCAM to assess the added value of urine ALCAM for discrimination in predicting patient survival. In addition, the continuous net reclassification index (NRI) was calculated using Uno's package [40].

Statistical analyses and graphing were performed with SPSS (IBM), GraphPad Prism (GraphPad Software, Inc.) and R V 3.1.0 (http://www.R-project.org) [50] and several R packages, including “Hmisc”, “rms”, “survivalROC” and “survIDINRI”. Bootstrap validation was performed via SPSS with a two-tailed significance, 1000 iterations and a Mersenne twister of 2,000,000 as well as in R as already described.

### Impact

Beyond the initiating genetic event, cancer progression and metastasis is primarily controlled by alterations in the proteome. The cell migration machinery and its functional products not only contribute mechanistically to metastatic dissemination but also have the potential to serve as markers of invasive disease. While ALCAM has been postulated as such a biomarker, multiple studies have yielded contradicting results. The current study utilizes gene expression, immunofluorescence staining, and ELISA analysis of serum and urine to demonstrate that ALCAM shedding, but not expression, corresponds to patient outcome in bladder cancer. Furthermore, this multi-institutional cohort analysis reveals that shed urinary ALCAM is an independent prognostic indicator of overall survival in patients undergoing cystectomy. We are the first to suggest that urinary ALCAM can aid in the identification of high-risk patients and in directing intervention. This data highlights the significance of focusing on protein function and post-translational events in identification of novel biomarkers.

## SUPPLEMENTARY MATERIALS


