# Supplementary material for: Shed urinary ALCAM is an independent prognostic biomarker of three-year overall survival after cystectomy in patients with bladder cancer
Source: Oncotarget. 2016 Nov 24;8(1):722–41. doi: 10.18632/oncotarget.13546 (PMC5352192; doi:10.18632/oncotarget.13546)
Supplement: Supplementary file 1 [file oncotarget-08-722-s001.pdf]

Shed urinary ALCAM is an independent prognostic biomarker of three-year overall survival after cystectomy in patients with bladder cancer

Supplementary Materials

Assessment of stability and repeatability of shed ALCAM

The stability of ALCAM in serum and urine specimens was assessed by measuring ALCAM levels by ELISA before and after a prolonged freeze-thaw cycle. Samples were thawed at 4-degrees Celsius for three days, re-froze, and then re-thawed on ice just prior to use. Pre- and post-freeze-thaw ALCAM levels were assessed by calculating fold-change. The influence of catheterization on urine ALCAM levels was assessed by analyzing urine ALCAM in patient-matched clean catch and foley specimens collected just prior to and during surgery, respectively. A paired *t*-test was performed to detect any differences in the matched samples. Repeatability or inter-assay variability of the detection of urine ALCAM was assessed by repeating the ELISA on two separate aliquots over two runs separated by more than six months. The Spearman correlation and the slope of the linear regression curve were calculated to determine the inter-assay correlation, where a perfect correlation would have an R_s^2 and slope of 1. In addition, the inter-assay variation was assessed with the Bland-Altman difference as well as, the quartile coefficient of dispersion, a nonparametric version of the coefficient of variation (CV). The ratio of the two dispersion coefficients shows that the inter-assay variation is 4–12%.

REFERENCES

1. Harrell FE, Jr., Lee KL, Mark DB. Multivariable prognostic models: issues in developing models, evaluating assumptions and adequacy, and measuring and reducing errors. Statistics in medicine. 1996; 15:361–87.

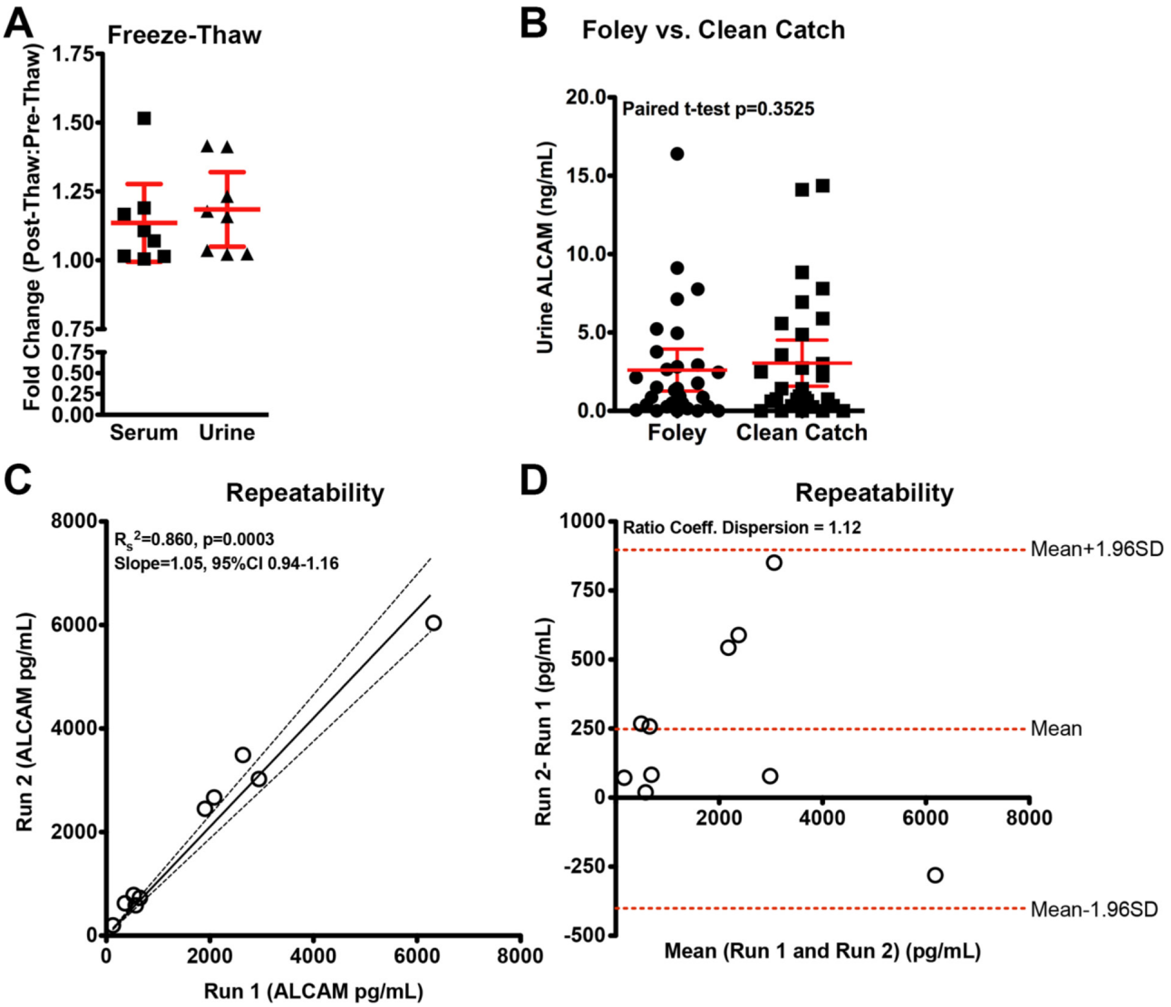

Supplementary Figure S1: Quality control for measuring shed ALCAM in fluids. (**A**) Analysis of the effect of freeze-thaw (3-day, 4-degree thaw) on levels of serum and urine ALCAM. Fold change reveals that there is no loss of detectable shed ALCAM in either serum or urine. Mean and 95% confidence intervals are graphed. (**B**) Comparison of ALCAM levels in matched clean catch versus foley-derived urine. Paired t-test is shown along with mean and 95% confidence intervals. (**C**, **D**) Assessment of the repeatability of urine ALCAM detection by ELISA. (C) Run 1 on the x-axis is plotted against Run 2 on the y-axis. Dashed lines are the upper and lower 95% confidence intervals. Spearman correlation (R_s^2) and slope are shown. (D) Bland-Altman plot of mean versus difference of run 1 and run 2. Red dashed lines indicate mean and 1.96 standard deviations (SD) above and below the mean. Quartile coefficient of dispersion, a nonparametric version of coefficient of variation (CV), is also calculated for each run and the ratio of the two coefficients determined to compare inter-assay variation. All analyses were two-tailed with a 0.05 significance level.

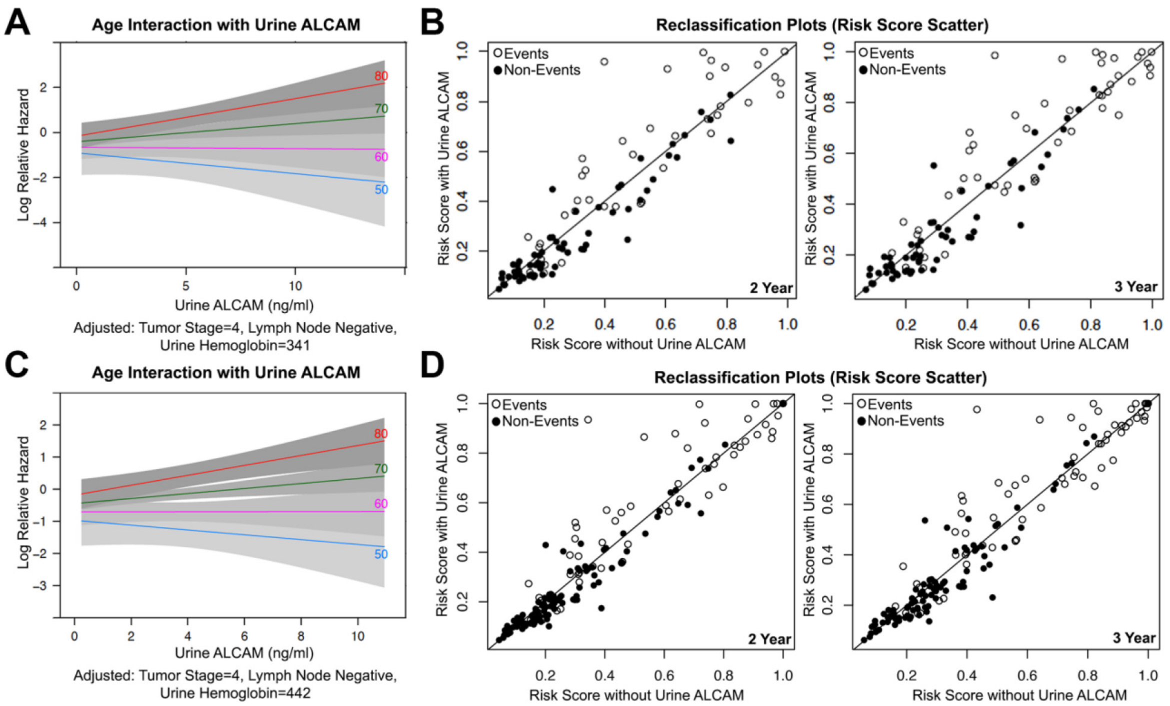

Supplementary Figure S2: Age interaction and prediction improvement in the multivariable model of 3-year overall survival in bladder cancer with the addition of urine ALCAM. (**A**, **C**) Graph of adjusted urine ALCAM effect for patients at ages 50, 60, 70 and 80 years old with stage 4, negative lymph node status, and urine hemoglobin at 341ng/ml for the VUMC cohort (A) or 442 ng/ml for the combined cohort (C). (**B**, **D**) Predicted risk of death for the VUMC cohort (B) and the combined cohort (D) at 2-year (left) and 3-year (right) for each patient from Model 2 against the predicted risk from Model 1 where events (open circles) and non-events (black circles) are displayed.

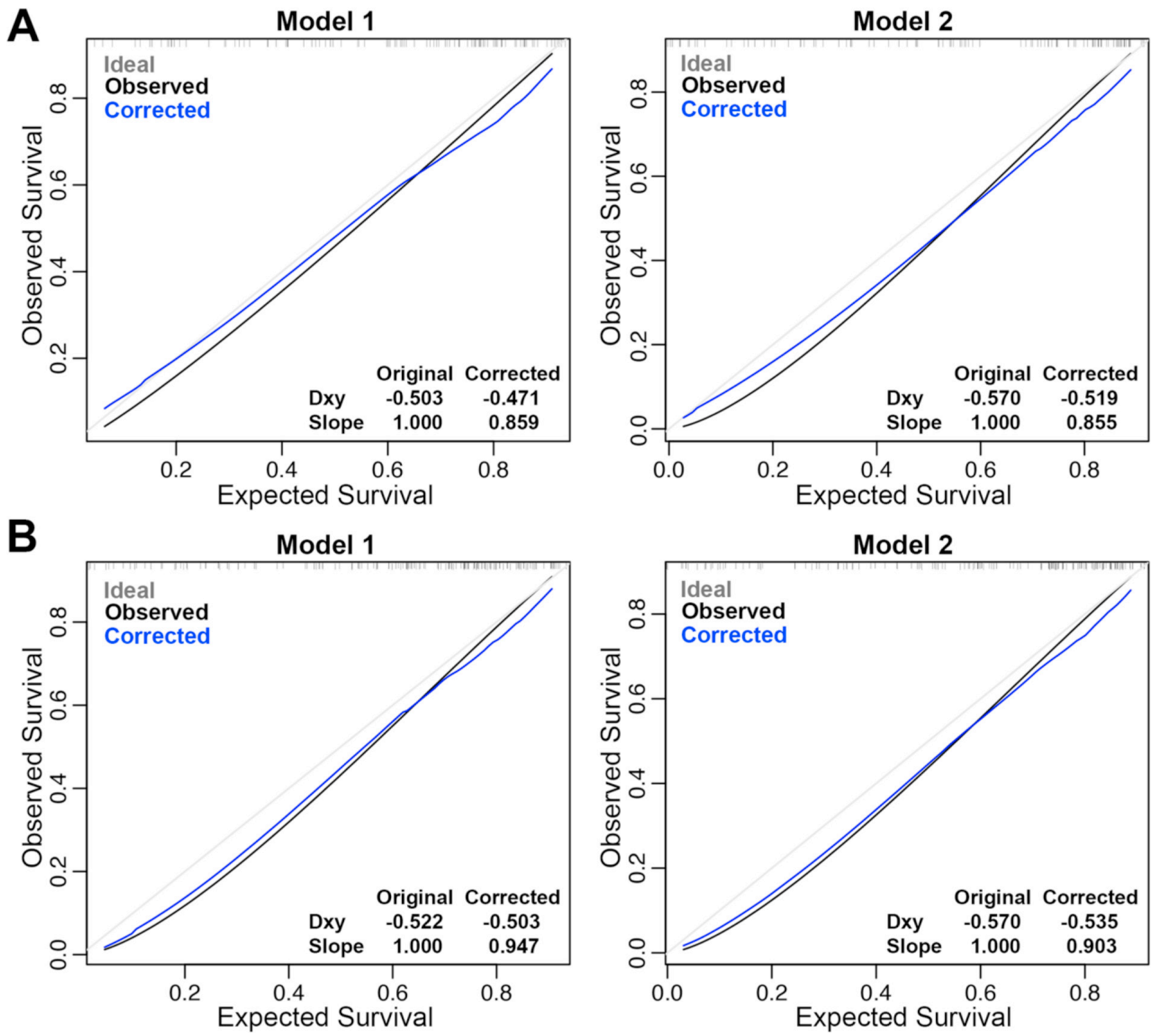

Supplementary Figure S3: Calibration of the models for 3-year overall survival and accuracy estimates using bootstrap. Adaptive linear spline hazard regression was used to draw the calibration curves, which are the observed fraction of survived against the predicted survival from the model. Black lines, observed curves; Blue lines, bootstrap (over-fitting) corrected curves. Model 1 (without urine ALCAM) and model 2 (with urine ALCAM) in the VUMC cohort (**A**), and the VUMC and UTSW combined cohort (**B**). Mean absolute error between observed and corrected estimates for (A) model 1 and 2 is 0.03 and 0.04 and (B) model 1 and 2 is 0.04 and 0.04, respectively. Harrell's internal validation (1) was used to assess over-fitting of model 1 and model 2 for prediction of 3-year overall survival. D_{xy}, Somer's D rank correlation between the predicted log hazard and observed survival time. Slope, shrinkage estimate for quantifying over-fitting. Corrected Index, values after bootstrap validation with 100 iterations.
